# Adenosine A_2A_ Receptor Blockade Prevents Rotenone-Induced Motor Impairment in a Rat Model of Parkinsonism

**DOI:** 10.3389/fnbeh.2016.00035

**Published:** 2016-02-29

**Authors:** Ahmed M. Fathalla, Amira M. Soliman, Mohamed H. Ali, Ahmed A. Moustafa

**Affiliations:** ^1^Faculty of Medicine, Department of Pharmacology, Suez Canal UniversityIsmailia, Egypt; ^2^Center for Aging and Associated Diseases, Zewail City of Science and TechnologyGiza, Egypt; ^3^Department of Veterans Affairs, New Jersey Health Care SystemEast Orange, NJ, USA; ^4^School of Social Sciences and Psychology and Marcs Institute for Brain and Behaviour, Western Sydney UniversitySydney, NSW, Australia

**Keywords:** Parkinson’s disease, dopamine, grid walk, stride length, rotenone, adenosine receptors

## Abstract

Pharmacological studies implicate the blockade of adenosine receptorsas an effective strategy for reducing Parkinson’s disease (PD) symptoms. The objective of this study is to elucidate the possible protective effects of ZM241385 and 8-cyclopentyl-1, 3-dipropylxanthine, two selective A_2A_ and A_1_ receptor antagonists, on a rotenone rat model of PD. Rats were split into four groups: vehicle control (1 ml/kg/48 h), rotenone (1.5 mg/kg/48 h, s.c.), ZM241385 (3.3 mg/kg/day, i.p) and 8-cyclopentyl-1, 3-dipropylxanthine (5 mg/kg/day, i.p). After that, animals were subjected to behavioral (stride length and grid walking) and biochemical (measuring concentration of dopamine levels using high performance liquid chromatography, HPLC). In the rotenone group, rats displayed a reduced motor activity and disturbed movement coordination in the behavioral tests and a decreased dopamine concentration as foundby HPLC. The effect of rotenone was partially prevented in the ZM241385 group, but not with 8-cyclopentyl-1,3-dipropylxanthine administration. The administration of ZM241385 improved motor function and movement coordination (partial increase of stride length and partial decrease in the number of foot slips) and an increase in dopamine concentration in the rotenone-injected rats. However, the 8-cyclopentyl-1,3-dipropylxanthine and rotenone groups were not significantly different. These results indicate that selective A_2A_ receptor blockade by ZM241385, but not A_1_ receptor blockadeby 8-cyclopentyl-1,3-dipropylxanthine, may treat PD motor symptoms. This reinforces the potential use of A_2A_ receptor antagonists as a treatment strategy for PD patients.

## Introduction

Parkinson’s disease (PD) is a progressive neurodegenerative disorder characterized by motor dysfunction (Kramberger et al., 2010). The loss of dopaminergic (DA) neurons is responsible for the development of PD motor symptoms (Liu, 2006). DA therapies, such as L-DOPA and dopamine agonists, either have a short half-life or may induce psychiatric side effects (Olanow et al., 2004; Moustafa et al., 2014). These issues raise the urgent need for an alternative form of therapeutic intervention.

Rotenone is a neurotoxin that replicates most of PD motor symptoms and leads toa loss of nigrostriatal DA neurons (Thiffault et al., 2000; von Wrangel et al., 2015). In this study, we used a rotenone model, as done in prior studies (Zaitone et al., 2012; Samim et al., 2014).

Adenosine is a neuromodulator in the striatum (Schiffmann et al., 2007), acting through four subtypes of G-protein coupled receptors, A_1_, A_2A_, A_2B_ and A_3_ receptors (Fredholm, 2010). A_2A_ receptors are co-localized with dopamine D_2_ receptors inthe indirect pathway of the basal ganglia (Morelli et al., 2007). The blocking of A_2A_ receptors causes locomotor activation by lowering the inhibitory function of the indirect pathway of the basal ganglia, which is similar to the effects of blocking dopamine D_2_ receptors activation (Jenner, 2014; Pinna et al., 2014). Thus, adenosine A_2A_ receptor antagonists are considered a promising strategy to treat PD (Schwarzschild et al., 2006; Pinna et al., 2014).

The adenosine A_1_ receptors are localized in the striatum presynaptically of dopamine axon terminals where they inhibit dopamine release (Borycz et al., 2007). ZM241385(4-(2-[7-amino-2-(2-furyl)[1,2,4]-triazolo[2,3-a][1,3,5]triazin-5-yl amino]ethyl) phenol) is an antagonist with high affinity at the adenosine A_2A_ receptor subtype in the brain (Cunha et al., 1997).

In this study, we test the protective effects of 8-cyclopentyl-1,3-dipropylxanthine as a selective A_1_ receptor antagonist and ZM241385 as a selective A_2A_ receptor antagonist in a rat model of PD induced by rotenone.

## Materials and Methods

### Animals

Thirty two adult male albino rats weighing 200 ± 20 g were used for the current study. Animals were purchased from the National Research Center for Experimental Animals, Cairo, Egypt. Animals were housed under standardized conditions away from any stressful stimuli with normal day/night cycle, 25 ± 2°C temperature, in plastic polyethelyne cages with free access to food and water and were permitted for acclimatization for 1 week before starting the study. The behavioral tests were conducted after rotenone injections at 4 p.m. to minimize circadian influence on behavior. All experimental protocols were approved by the Institutional Animal Care and Use Committee at Suez Canal University. All efforts were exerted to reduce animal suffering and to minimize the number of animals used.

### Drugs

ZM241385 and 8-cyclopentyl-1,3-dipropylxanthine (DPCPX) were purchased from Sigma-Aldrich (St. Louis, MO, USA) and dissolved in saline solution (Chen et al., 2001). ZM241385 or DPCPX were administered intraperitoneally (IP) at a dose of 3.3 or 5 mg/kg/day, respectively, for 12 consecutive days in a volume of 1 ml/kg (Chen et al., 2001).

Rotenone was purchased from Sigma-Aldrich (St. Louis, MO, USA) and dissolved in 1:1 (v/v) dimethylsulfoxide (DMSO) and polyethyleneglycol (PEG-300; Thiffault et al., 2000). Rats received six subcutaneous injections of rotenone (1.5 mg/kg/48 h, s.c.) in a volume of 1 ml/kg. The rotenone-treated animals showed signs of akinesia and rigidity starting from the third injection (Thiffault et al., 2000).

ZM241385 and 8-cyclopentyl-1,3-dipropylxanthine (DPCPX) were purchased from Sigma-Aldrich (St. Louis, MO, USA) and dissolved in a saline solution (Chen et al., 2001). ZM241385 or DPCPX were administered IP at a dose of 3.3 or 5 mg/kg/day, respectively, for 12 consecutive days in a volume of 1 ml/kg (Chen et al., 2001).

### Study Design

Rats were randomly divided into four groups, each has eight animals: (a) (vehicle-control group): rats received six intraperitoneal injections of the vehicle in a volume of 1 ml/kg; (b) (rotenone group): rats received subcutaneous rotenone (1.5 mg/kg/48 h) and received normal saline in a volume of 1 ml/kg daily for 12 days; (c) (ZM241385-treated group): 10 min before rotenone injection, rats received daily doses of intraperitoneal ZM241385 at a dose of 3.3 mg/kg daily for 12 days; and (d) (8-cyclopentyl-1,3-dipropylxanthine-treated group): 10 min before rotenone injection, rats received daily doses of intraperitoneal 8-cyclopentyl-1,3-dipropylxanthine at a dose of 5 mg/kg daily for 12 days.

### Tasks and Functional Assessment

Rats were screened for motor impairment using the stride length and grid walking tests.

#### Stride Length Quantitative Gait Analysis Test (Fernagut et al., 2002)

Rats were habituated to the apparatus for 3 days before the beginning of the experiment. The apparatus was composed of an open field (60 × 60 × 40 cm) illuminated by a light, in which a runway (4.5 cm wide, 42 cm long, borders 12 cm height) was prepared to lead out into a dark wooden box (20 × 17 × 10 cm). Stride length was measured by wetting animal fore- and then hind-paws with black ink; animals were then allowed to trot on a paper strip (4.5 cm wide, 40 cm long) down the brightly lit runway towards the dark goal box. First, the length of the forelimb stride was measured in all animals, followed by the hind-limbs on a new strip of paper, directly after drying of the forelimb inked paws. The manual measurement of stride length was performed as the distance between two paw-prints. The mean of the longest three of the measured stride length (corresponding to maximal velocity) were measured in each run. We excluded paw-prints made at the beginning (7 cm) and the end (7 cm) of the run due to velocity changes. Any runs in which the rats stopped or made an obvious decelerations observed by the experimenter were excluded from analysis.

#### Grid Walking Test (Menet et al., 2003)

This test assesses the ability of accurate placing the forepaws during spontaneous exploration of an elevated grid by calculating the frequency of failure to accurately hold the rungs. Here, rats were placed on a wire grid (330 mm in diameter with 15 × 15 mm grid squares) and allowed to freely move for 3 min. The rats were videotaped and subsequently an experimenter blinded to the treatment group scored the number of foot slips in the first 50 steps, with the left and right fore- and hind-paws. A foot slip was recorded either when the paw completely fails to hold a rung, thus the limb dropped in between the rungs, or when the paw was accurately placed on the rung but fell during weight bearing. No pre-training of animals was required but they were put on the grid twice prior to injection for habituation and to obtain baseline scores.

### Brain Tissue Preparation for Measuring Dopamine Levels in the Midbrain

At 4 p.m. of the following day (24 h after the last assessment of motor performance), rats were anesthetized by injection of thiopental sodium (30 mg/kg, intraperitoneal; Flecknell, 1993) and sacrificed by decapitation. Their brains were quickly dissected, the midbrain was surgically dissected and washed with ice-cold saline, and then weighed and rapidly frozen (−80°C of liquid nitrogen) until used for determination of dopamine by high performance liquid chromatography (HPLC) according to the method of Hussein et al. (2012). Frozen tissues were cut into small pieces and homogenized in phosphate buffer (pH 7.4), then centrifuged at 4000 rpm for 15 min at 4°C to spin down tissue fragments, nuclei and mitochondria. The supernatant was removed and filtered through a 0.2 micrometer teflon syringe filter for HPLC analysis. The measurement of dopamine levels was carried out by using a HPLC system, Agilent technologies 1100 series, equipped with an aquaternary pump (Quat pump, G131A model). The separation of dopamine was carried out by means of ODS-reverse-phase column (c18, 25 × 0.46 cm i.d. 5 μm). The mobile phase consists of 50 mM potassium phosphate buffer/methanol 97/3 (v/v), pH 3.5 and was delivered at flow rate of 1.5 ml/min. The substrates were detected by UV at 270 nm. The injection volume was 20 microliter. Serial dilutions of dopamine HPLC standard were injected, followed by a determination of their peak areas. A linear standard curve was drawn by plotting peak areas vs. the corresponding concentrations. The concentration of samples was obtained from Hussein et al. (2012).

### Statistical Analysis

Data were expressed as mean ± SEM and analyzed using the statistical package of social sciences (SPSS program, version 17, SPSS Inc., Chicago, IL, USA). The assessment of difference of mean values among groups was conducted using one-way analysis of variance (ANOVA) followed by Bonferroni’s multiple comparisons test. *p* < 0.05 was considered significant.

## Results

### Behavioral Results

#### Stride Length Quantitative Gait Analysis Test

**Figure 1:**
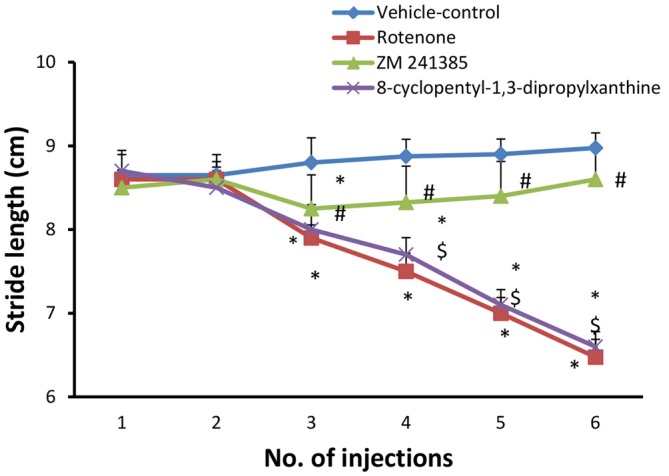
**Stride length test in the experimental groups.** The figure shows forelimbs and hindlimbs stride length (cm) in the experimental groups. Rotenone induced a significant difference in the mean stride length between the forelimbs and hindlimbs, while ZM241385 improved it significantly and 8-cyclopentyl-1,3-dipropylxanthine did not improve it compared to the rotenone group. Data were expressed as mean ± SE, analyzed using one way analysis of variance (ANOVA) followed by Bonferroni *post hoc* test, *n* = 8. *p < 0.05 compared to the vehicle-control group, ^#^*p* < 0.05 compared to rotenone group, ^$^*p*< 0.05 compared to the ZM241385-treated group.

Systemic administration of rotenone (1.5 mg/kg, s.c. every other day, 6 doses) produced a significant difference between the stride length of forelimbs and hindlimbs starting from the third injection (Figure 1, *p* < 0.05). After the last injection, the mean of longest three of the measured stride was (6.47 ± 0.213 cm) in the in the rotenone group and (8.97 ± 0.60 cm) in the vehicle-control group. Compared to rotenone, ZM241385 significantly increased the stride length of forelimbs and hindlimbs of rats (8.6 ± 0.41 cm; Figure 1, *p* < 0.05), whereas 8-cyclopentyl-1,3-dipropylxanthine was devoid of effects (6.6 ± 0.17 cm; Figure 1, *p* > 0.05).

#### Grid Walking Test

**Figure 2:**
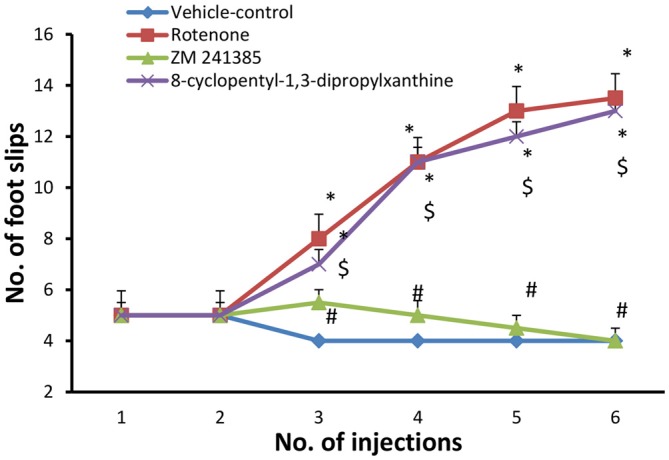
**Grid walking test in the experimental groups.** Here, we show the number of foot slips in the experimental groups in the grid walking test. Rotenone induced higher foot slip errors compared to vehicle-control group starting from the third injection. The treatment with ZM241385 decreased the number of foot slip errors compared to the rotenone group. Data were expressed as mean ± SE, analyzed using one way ANOVA followed by Bonferroni *post hoc* test. *n* = 8. *p < 0.05 compared to vehicle-control group, ^#^*p* < 0.05 compared to rotenone group, ^$^*p* < 0.05, compared to the ZM241385-treated group.

The number of foot slips in the first 50 steps was measured. In the present study, grid walking test was performed after each injection with either vehicle (group 1) or rotenone (groups 2, 3 and 4). when compared to the vehicle-control group (4 ± 0.001), rotenone significantly (*p* < 0.05) increased the number of foot slips beginning from the third injection (13.5 ± 0.95) throughout the study. Compared to the rotenone group, ZM241385 decreased in a sustained and significant manner the number of foot slips (4 ± 0.5 at third injection), whereas DPCPX was devoid of effects (13 ± 0.57 at third injection) compared to rotenone group (Figure 2, *p* > 0.05).

### Dopamine Level Analysis Results

**Figure 3:**
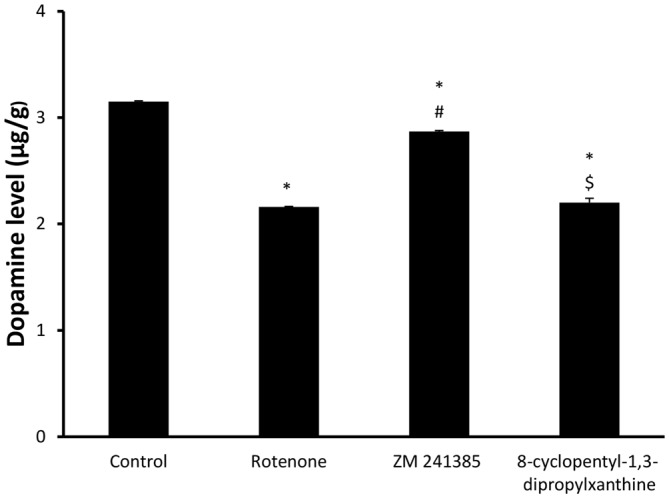
**Dopamine levels in all four groups.** Here, we show DA concentration in the different experimental groups. The administration of rotenone resulted in a significant decrease in DA levels, while ZM241385 increased DA levels, in comparison to the rotenone group. Data were expressed as mean ± SE, analyzed using one way ANOVA followed by Bonferroni *post hoc* test, *n* = 8. *p < 0.05 compared to the vehicle-treated group, ^#^*p* < 0.05 compared to the rotenone group, ^$^*p* < 0.05 compared to the ZM241385-treated group.

Dopamine levelsin the midbrain in the vehicle-control group were 3.15 ± 0.02 μg/g wet tissue and were significantly (*p* < 0.05) reduced to 2.16 ± 0.01 μg/g wet tissue by rotenone. ZM241385 significantly (*p* < 0.05) attenuated the impact of rotenone on dopamine levels in the midbrain (2.87 ± 0.02 μg/g wet tissue), whereas DPCPX was devoid of effects (2.2 ± 0.081 μg/g wet tissue; Figure 3, *p* > 0.05).

## Discussion

Our results demonstrate that rotenone-treated rats exhibited motor deficits in the stride length and grid walking tests, as described by others (Hisahara and Shimohama, 2010; Li et al., 2012; von Wrangel et al., 2015), as well as lower dopamine levels in the midbrain (Höglinger et al., 2003; Sharma and Nehru, 2013), supporting its validity as a PD model. Notably, the A_2A_R antagonist ZM241385 attenuated all these alterations induced by rotenone, whereas the A_1_ receptor antagonist, DPCPX was devoid of effects. These findings, using a different animal model of PD and different behavioral tests of motor function, re-enforce the benefits afforded by A_2A_ receptor blockade in different tests and animal models of PD (reviewed in Schwarzschild et al., 2006; Pinna et al., 2014), which are not mimicked by A_1_ receptor antagonists (Chen et al., 2001). This efficiency of A_2A_ receptors to control motor dysfunction in PD, probably result from the ability of A_2A_R to control a series of concurrent processes, such as the release of glutamate from corticostriatal terminals that engage striatal circuits (Quiroz et al., 2009), the processing of information by medium spiny striatal neurons (Higley and Sabatini, 2010; Shen et al., 2013), the control microglia reactivity and neuroinflammation (Gyoneva et al., 2014) and the astrocytic support of neuronal function (Matos et al., 2015), the control the trophic support of DA terminal in the striatum (Gomes et al., 2009), the loss of nerve terminals and apoptosis of neurons (Silva et al., 2007), as well as the aggregation of α-synuclein (Ferreira et al., 2015).

Our study is not without limitations. First, future histopathological studies should investigate the effects of A_2A_ receptors blockade on the levels of dopamine metabolites to confirm or disconfirm our findings. Second, additional experimental studies are needed to explore the possible preventive and curative molecular mechanisms of adenosine A_2A_ receptors antagonists. Also, additional long-term studies with a large sample size should be carried out for further assessment of the effects of long-term duration of adenosine A_2A_ receptor antagonists on different PD models. Finally, as this is a pharmacological study, it is assumed that our results are related to adenosine antagonism, based on prior findings (Cunha et al., 1997).

## Author Contributions

Study was conducted by AMF and AMS. Writing was done by all co-authors.

## Conflict of Interest Statement

The authors declare that the research was conducted in the absence of any commercial or financial relationships that could be construed as a potential conflict of interest.
